# The hospital management practices in Chinese county hospitals and its association with quality of care, efficiency and finance

**DOI:** 10.1186/s12913-021-06472-7

**Published:** 2021-05-11

**Authors:** Yidan Zhu, Yifei Zhao, Lixia Dou, Ruya Guo, Xuefei Gu, Runlin Gao, Yangfeng Wu

**Affiliations:** 1 Peking University Clinical Research Institute, Beijing, China; 2 Department of Epidemiology and Biostatistics, School of Public Health, Peking University, Rm.106, Bldg.6, No.38 Xueyuan Rd., Haidian Dist, Beijing, China; 3 China National Health Development Research Center, Beijing, China; 4 The Department of Cardiology, Fuwai Hospital, Chinese Academy of Medical Sciences and Peking Union Medical College, Beijing, China; 5 The George Institute for Global Health at Peking University Health Science Center, (PUHSC), Beijing, China

**Keywords:** Hospital management practice, County hospital, Quality of care, Efficiency, Finance, China

## Abstract

**Background:**

County hospitals as the backbone of the China’s healthcare system are providing services for over 70% of the total population. However, the hospital management practice (HMP) and its links with quality of care, efficiency and finance in these hospitals are unknown.

**Methods:**

We did two cross-sectional surveys of HMP in 2013 and 2015 among 101 county hospitals across rural China. Three managing roles (hospital director, director of medical affairs office and director of cardiology) and a cardiologist were invited to the surveys. A novel HMP rating scale, with 100 as full score, was used to measure the HMP in 17 indicators under four dimensions (target, operation, performance, and talent management) for each hospital. We analyzed the association of HMP score with variables on quality of care, efficiency and finance using linear mixed models with and without adjustment for potential confounders.

**Findings:**

A total of 95 hospitals participated in at least one survey and were included in the analysis. The overall mean HMP score varied dramatically across the hospitals and 84% of them scored less than 60. The dimension mean HMP score was 38.6 (target), 56.4 (operation), 53.2 (performance) and 55.7 (talent), respectively. The pattern of indicator mean HMP score, however, was almost identical between hospitals with high and low overall HMP score, showing the same ‘strength’ (staff satisfaction, staff performance appraisal, ‘hard wares’, patient-centered services, etc.) and ‘weakness’ (target balance, target setting, continuous quality improvement, penalties on staff with dissatisfied performance, etc.). The associations of overall mean HMP score with quality of care and efficiency variables and cost per hospitalization was not statistically significant. The statistical significance in the association with hospital annual total income disappeared after adjusting for region, teaching status, number of competitors, number of staff and number of beds in use.

**Conclusion:**

The HMP in Chinese county hospitals scores low in general and was not significantly associated with hospital care quality, efficiency and finance. The current healthcare reform in China should address the micro level issues in hospital management practices.

**Supplementary Information:**

The online version contains supplementary material available at 10.1186/s12913-021-06472-7.

## Background

According to the 2018 statistics, there were 12,109 public hospitals in China, and more than half of them were county hospitals [1]. County hospital serves as the highest level hospital in the three-tier healthcare network (i.e. county hospitals – township hospitals/health stations – village clinics) in a county, an administrative region of rural China. These hospitals are secondary mostly and tertiary in few cases. As the backbone of the country’s health care system, county hospitals provide health care services to more than 900 million residents in China, accounting for over 70% of the total population [2]. The current healthcare reform in county hospitals started in 2012 [3] to enhance hospital capacity for better rural health services through improving rural health financing, quality, and efficiency in service delivery as well as hospital management. The reform was expanded to more than a thousand county hospitals in 2014 [4], and further to all the county hospitals in 2015 [5]. The task was to establish a modern hospital management system to improve hospital management practices, and that should be achieved by enhancing leadership, optimizing administration and operation system, establishing reasonable performance appraisal system and improving the mechanism of staff motivation and salary system [6].

Evidence showed that the improvement of the hospital management system can effectively strengthen hospital competitiveness, and improve medical care quality and patient satisfaction [7, 8]. Tsai and his colleagues [9] found that higher management performance and effective board were related to higher healthcare quality. McConnell KJ [10] found an 8% increase in hospitalization rate of patients with acute myocardial infarction was related to the hospital management improvements. Besides, a number of large prospective observational studies showed that performance management can improve medical staff’s compliance to guidelines and the rate of patients receiving guideline-recommended drugs [11, 12]. For example, appropriate staff incentives were linked to an increase in aspirin used for secondary prevention from 87 to 95% and beta blockers from 81 to 93% in patients with acute coronary syndromes [11].

However, there has been no study to help us understand the current status of hospital management practices in Chinese hospitals and whether these practices are linked to core measures of hospital performance. In this study, we applied a hospital management practice rating scale [13] to describe the current status of HMP in Chinese county hospitals and the associations of HMP with hospital quality of care, efficiency, and finance.

## Methods

### Study hospitals

This study is part of the ancillary study of the Third Phase of the Clinical Pathways for Acute Coronary Syndromes in China (CPACS-3) Program [14, 15], a stepped-wedge cluster-randomized trial conducted from 2011 to 2015 to evaluate a multifaceted quality of care improvement intervention program among ACS patients in 101 county hospitals from 15 provinces of China. The ancillary study was to develop a HMP rating scale, to quantify the hospital management practices, and to examine the associations between HMP and quality of care among CPACS-3 study participating hospitals [13]. The ancillary study included two cross-sectional surveys conducted first in 2013 and repeated in 2015. Hospitals that participated in at least one survey were included in the present analysis. The study was approved by the Peking University Institutional Review Board.

### Data collection

The hospital management survey was carried out twice in 2013 and 2015 respectively to collect data for calculating HMP score. The surveys used self-designed questionnaires to collect information from four hospital staff members with different roles in each participating hospital, including the hospital director (or deputy director), the director of medical affair management office, the chair and a cardiologist of the cardiology department. In addition, we also collected four types of hospital management documents including the long-term plan, annual action plan, performance appraisal policy and health care quality assurance policy. To ensure the quality of data collection, questionnaires were distributed and received in independent and sealed envelopes. All investigators and hospital coordinators were uniformly trained.

### Measurement of hospital management practices

We used the CPACS-3 Hospital Management Practice Measurement Instrument (the HMP scale) to quantify the level of HMP of each participating hospital. The scale is a novel rating scale developed by our study group and had been published previously [13]. In brief, the scale is based on data collected from the questionnaires and hospital management documents and it covers 4 dimensions (target management, operation management, performance management, and talent management) of hospital management practices and 17 indicators. Each indicator is given a score of 1. Of the four dimensions, the full scores of target management, operations management, performance management and talent management are 4, 5, 5 and 3 respectively. The overall management score is the summation of the four-dimension scores with the maximum value of 17. All scores used in this study were transformed into a 0–100 scale for analysis. For example, the overall HMP score was calculated by dividing the original score by 17 and multiplying by 100, so that readers could easily judge the goodness of a hospitals’ management practices with common sense. The HMP framework and definitions for indicators are listed in Supplementay Table S1.

### Measurement of quality of care, efficiency and finance

Hospital quality of care, efficiency, and finance were measured according to information collected from the questionnaire of the medical affair management office in each hospital. Indicators for quality of care included in-hospital death rate and nosocomial infection rate. Indicators for hospital efficiency included patient hospital stay, bed turnover times per year and hospital bed occupancy rate. Indicators for hospital finance included annual hospital total income and cost per hospitalization. All indicators reflect the annual data of the hospital.

### Covariates

Covariates were hospital-level characteristics including region, teaching status, number of competitors, number of staff and number of beds in use. Competitors was defined as other hospitals at the same technical level of medical services and in the same county. Staff referred to all employees working in the hospital including medical, administrative and support staff.

### Statistical analysis

Paired t-Test was performed to assess differences in mean overall HMP scores between year 2013 and 2015 for hospitals participated in both surveys. Since there was no significant difference between the 2 years, we used the two-year average score to classify the hospitals into two groups (low- and high- performance hospitals). For hospitals participated in only one survey, the HMP score of the year was used. We then compared each HMP indicator score between the two groups, in order to understand the differences between two groups. Bonferroni correction [16] was used to adjust the *p*-value to 0.003 for statistical significance to avoid false positive results due to multiple comparisons.

We used linear mixed models to examine the associations of HMP score with quality of care, efficiency and finance, in order to maximize the use of all available data at both surveys and better handle missing data. Adjusted mean difference between low- and high- performance groups and its 95% confidence intervals (CIs) were reported. We further adjusted for region, hospital teaching status, number of local competitors, number of hospital staff and number of beds in use. A two tailed *P* value of 0.05 was considered statistically significant. Analyses were performed using SAS, version 9.4 (SAS Institute, Cary, NC).

## Results

Among the 101 eligible hospitals, 86 hospitals participated in the baseline survey in 2013 and 92 hospitals participated in the second survey in 2015. A total of 95 hospitals that completed at least one survey were included in the present study. The baseline characteristics of the participating hospitals were shown in Table 1.

**Table 1:** Characteristics of the 95 participating hospitals^a^

Hospital characteristics	Total (*N* = 95)	Low HMP hospitals (*N* = 48)	High HMP hospitals (*N* = 47)	*P* value
Location^b^, n (%)				
East	33 (34.7)	11 (25.0)	18 (44.7)	0.024
Central	42 (44.2)	17 (43.8)	20 (44.7)	
West	20 (21.1)	15 (31.3)	5 (10.6)	
Teaching hospital, n (%)	73 (76.8)	34 (70.8)	39 (83.0)	0.161
Number of competitors, n (%)				
0	38 (41.3)	18 (38.3)	20 (44.4)	0.648
1	33 (35.9)	19 (40.4)	14 (31.1)	
≥ 2	21 (22.8)	10 (21.3)	11 (24.4)	
Number of staff, mean ± SD	668 ± 281	580 ± 235	758 ± 297	0.002
Number of beds in use, median (IQR)	585 (360, 800)	500 (287, 698)	650 (440, 900)	0.009

Data are shown as number (%), mean ± SD or median (IQR)

^a^Data reported here are mainly based on the first survey in 2013. For the nine hospitals that only participated in the second survey in 2015, data from second survey are used

^b^The local economy is highly associated with its geographic location in China, it is usually more developed in the east and less developed in the west

### The current status in HMP in participating hospitals

**Fig. 1:**
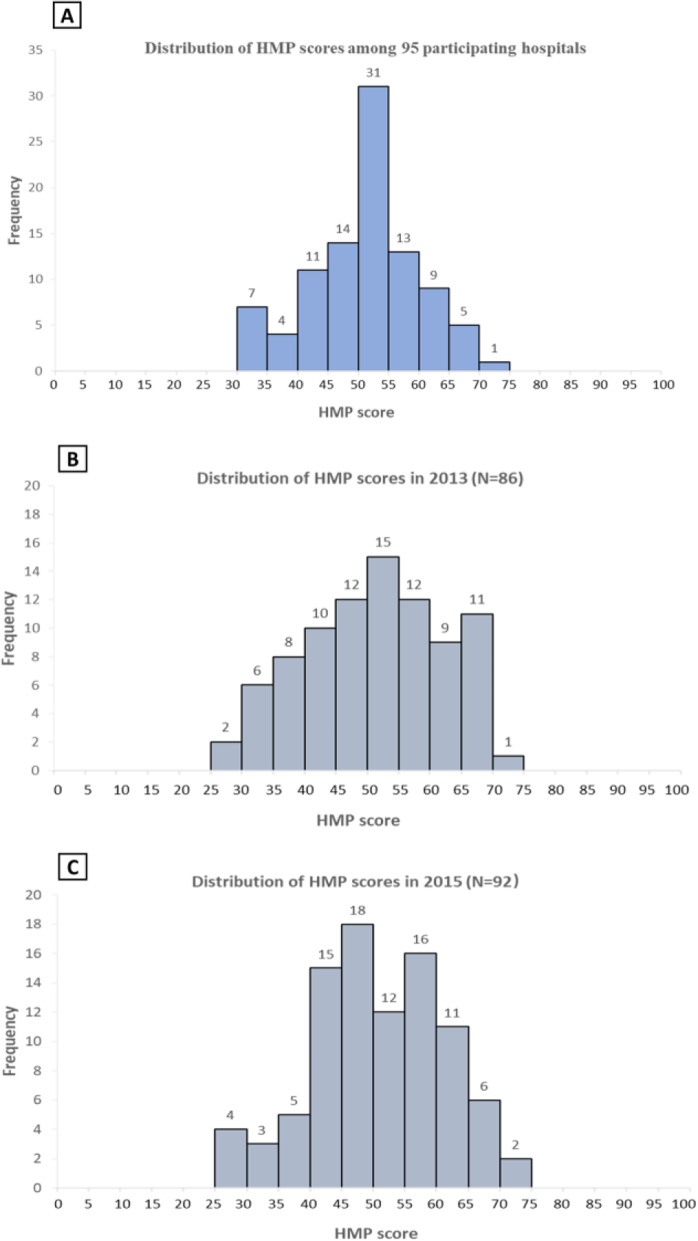
Distribution of HMP score (A: average score; B: score in 2013; C: score in 2015). Panel **a** shows distribution of overall HMP score (two-year average) among 95 hospitals participated in at least one survey, panel **b** and panel **c** show distribution of overall HMP score in 2013 and in 2015 respectively. Higher HMP scores represent higher hospital management practices

**Fig. 2:**
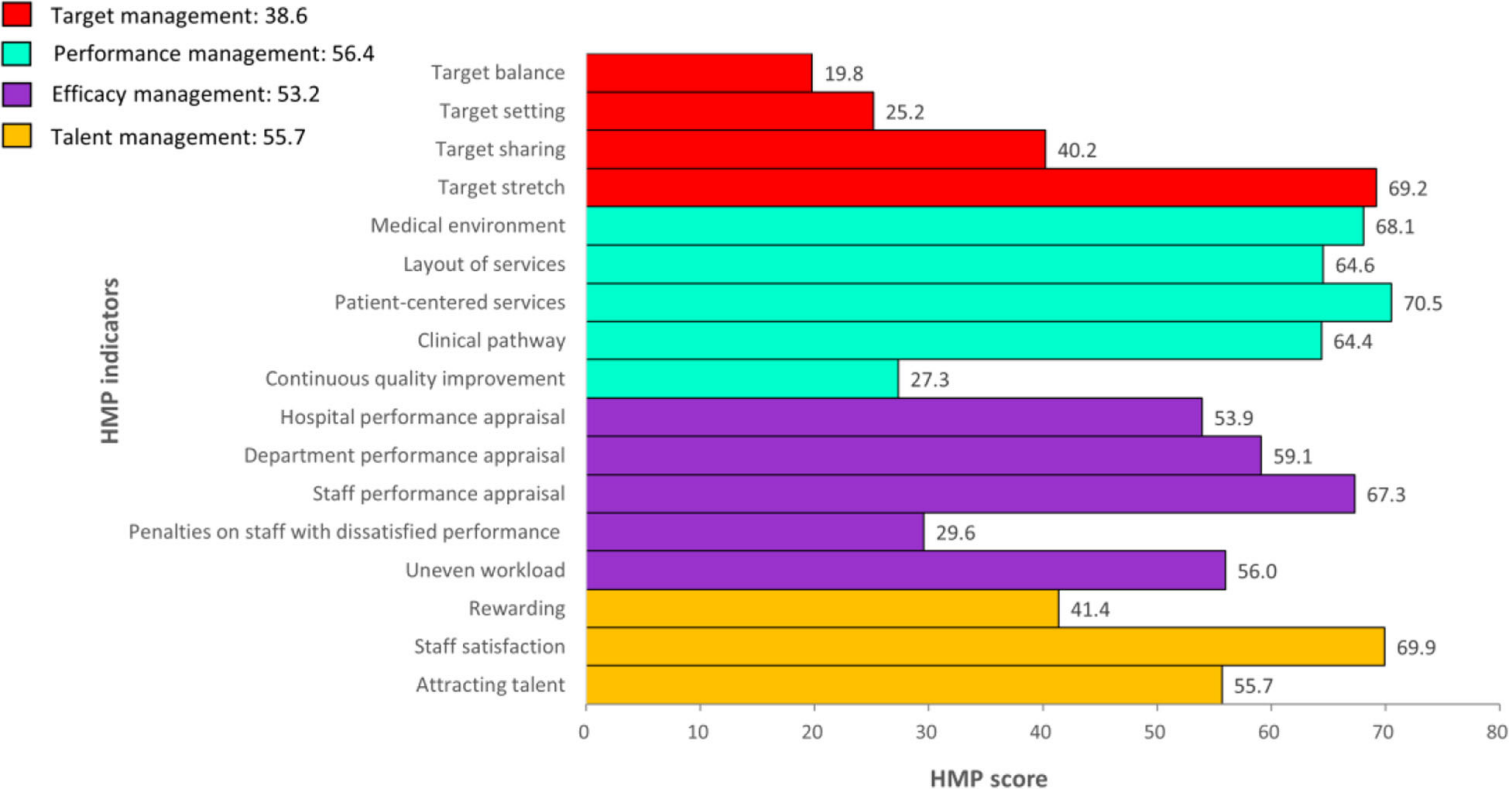
Mean HMP score for each dimension and indicator among 95 hospitals. The HMP score for each dimension and indicator ranges from 0 to 100, higher HMP scores represent better management practices

The distribution of HMP scores are shown in Fig. 1. Among 95 participating hospitals, the mean overall HMP score was 51.1 and ranged from 71.1 at the highest to 31.1 at the lowest, and 84% of hospitals scored less than 60 out of 100. The four dimensions of target management, operation management, performance management as well as talent management each had a mean score of 38.6, 56.4, 53.2 and 55.7 respectively (Fig. 2).

Among all indicators, the mean scores on “Target balance” and “Target setting” were the lowest, the mean scores on “Continuous quality improvement” and “Penalties on staff with dissatisfied performance” were only higher than the former two indicators, and the mean scores on “Target sharing” and “Rewarding” were also lower than the overall HMP mean score, i.e. 51.1.

### Comparison of the indicator scores between hospitals with high and low HMP scores

**Fig. 3:**
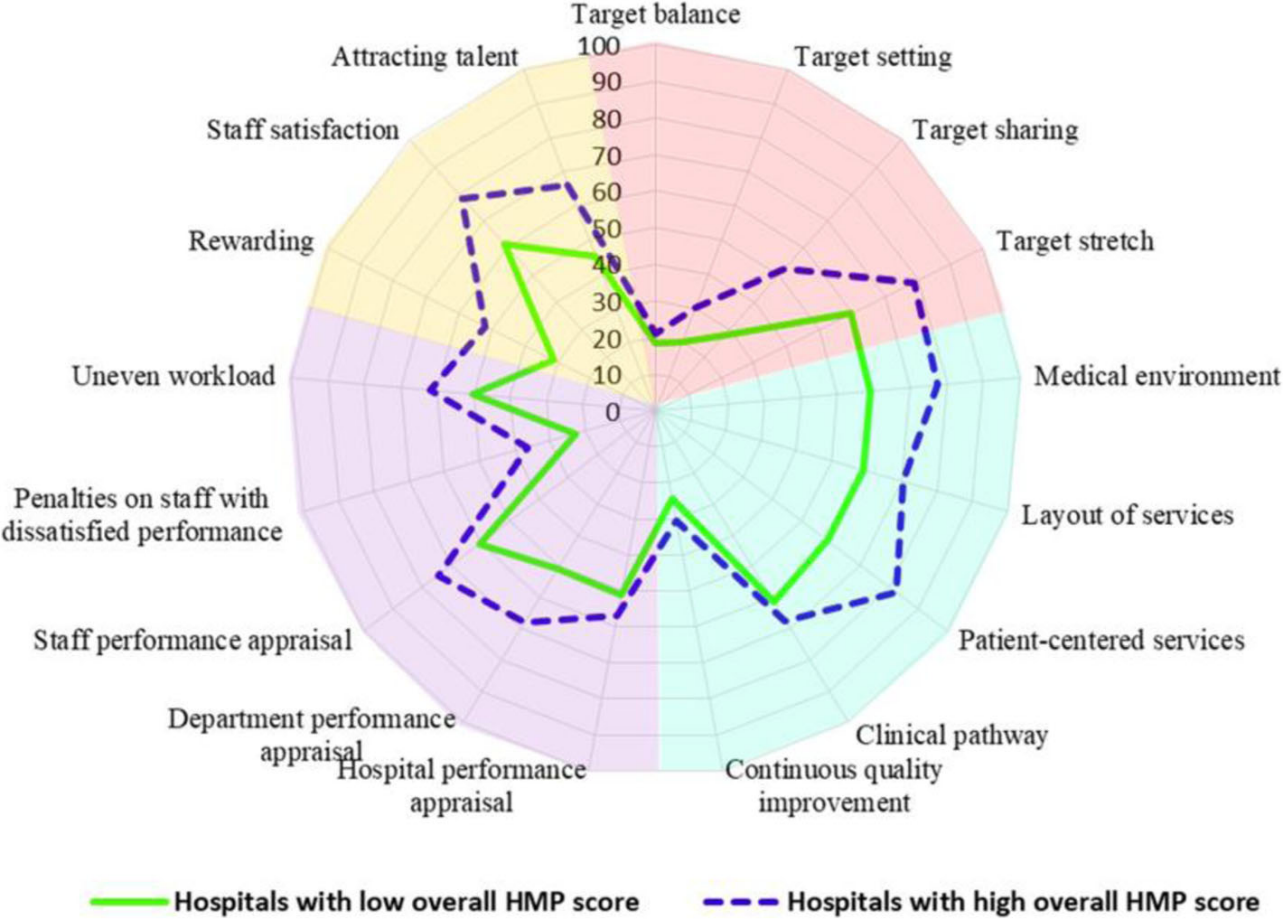
Radar chart of the indicator scores for hospitals with high and low HMP. For the 17 indicators, 11 indicators were significantly different (*P* < 0.003) between the two groups except for the “Target balance”, “Target setting”, “Layout of services”, “Clinical pathway”, “Continuous quality improvement”, and “Hospital performance appraisal”

To understand the possible differences in specific aspects of HMP between hospitals with high and low HMP scores, we plot the mean indicator scores by HMP groups on a radar map (Fig. 3). Interestingly, the shape or pattern of the radar map for the two groups were almost identical, where the indicators scored high (or low), in relation to other indicators, in high HMP group also scored high (or low) in low HMP group. Furthermore, the differences between the two groups of hospitals were mainly found in indicators with higher scores rather than indicators with lower scores. In fact, the “Target balance” and “Continuous quality improvement” had almost identical mean score between groups.

### Associations of HMP score with variables in quality of care, efficiency, and finance

As shown in Table 2, in the crude model, high HMP scores were significantly associated with higher annual total income but not with variables in quality of care and efficiency, and cost per hospitalization. After adjustment for region, hospital teaching status, number of competitors, number of staff and number of beds in use, HMP scores was no longer associated with hospital annual total income; the associations with other variables were not statistically significant too.

**Table 2:** HMP and its association with variables in quality of care, efficiency and finance

Outcomes	Hospitals with low HMP scores^a^	Hospitals with high HMP scores^a^	Crude Model^b^		Adjusted Model^c^	
			Difference (95%CI)	*P*	Difference (95%CI)	*P*
**Hospital care quality**						
In-hospital death rate(‰)	5.7 (0.9)	5.6 (0.9)	−0.1 (−2.6, 2.3)	0.914	−1.1 (−3.5, 1.4)	0.401
Nosocomial infection rate(‰)	8.3 (1.3)	9.5 (1.3)	1.2 (−2.0, 4.3)	0.457	0.3 (−3.2, 3.9)	0.846
**Hospital efficiency**						
Patient hospital stay (day)	8.3 (0.2)	8.2 (0.2)	−0.1 (−0.8, 0.5)	0.702	−0.2 (− 0.9, 0.5)	0.619
Bed turnover times per year	40.7 (1.7)	39.1 (1.7)	−1.6 (−6.3, 3.2)	0.518	−1.3 (− 6.6, 4.1)	0.632
Hospital bed occupancy rate (%)	96.5 (2.4)	98.4 (2.4)	1.9 (−4.6, 8.5)	0.563	1.7 (−4.8, 8.2)	0.602
**Hospital finance**						
Annual hospital total income (million USD^d^)	22.5 (0.2)	29.0 (0.2)	7.2 (0.6, 12.4)	0.032	0.1 (−3.9, 4.2)	0.954
Cost per hospitalization (USD)	653.6 (33.0)	724.4 (32.1)	70.9 (−20.7162.4)	0.128	17.7 (− 102.8, 67.3)	0.679

^a^Adjusted HMP scores with time fitted as random effects are shown as mean (SE)

^b^Linear mixed model with intercept and time fitted as random effects.

^c^Covariates adjusted included region, hospital teaching status, number of competitors, number of staff and number of beds in use.

^d^The current exchange rate of RMB to US Dollar in 2015 is 6.5.

## Discussion

In this study of 95 county hospitals in China, we found that the HMP in these Chinese county hospitals was generally poor, with 84% them scored less than 60 out 100 and with no evident change between the year 2013 and 2015. More important, we found the HMP scores were not statistically significantly associated with quality of care and efficiency but associated with the total income. However, the association with total income disappeared after the adjustment for possible mediators and confounders such as economic region and number of beds.

Our results support the media reports frequently seen on the newspapers that hospitals in China are pursuing money returns through the services provided [17], which is understandable due to the insufficient public funding to the hospitals [18, 19]. The problem is why the HMP score is not associated with the quality of care and the services efficiency.

Previous studies [9, 10] have reported the association between management performance and quality of care. A study of 103 hospitals in the United States and England [9] measured HMP in aspects of operation, monitoring, targets and human resources and found that higher HMP was associated with better quality of care. Contrary to the previous studies, we found that the association of HMP with quality of care in Chinese rural county hospitals was not statistically significant. Although the relatively small sample size may be an explanation, the executives of these hospitals have not really paid attention to the quality of care is certainly another explanation. This argument is well supported by our following observations in the study. In the dimension of target management, only 37% of our study hospitals included quality of care in the targets set and around half have no detailed action plan to improve the quality of care. In operation management, around two third of the hospitals reported not having “Continuous quality improvement” initiatives, indicating this important hospital management practice was lacking in most of the county hospitals in China. In the dimension of performance management, only 9% of the hospitals issued harsh penalties, such as removing from original post and deduction of bonus to staff with dissatisfied performance. In the dimension of talent management, less than half hospitals would reward the talents.

Interestingly, we observed a very similar pattern of management practices on the 17 indicators between hospitals with high and low HMP performances. Furthermore, the difference in overall HMP score between the two groups of hospitals were seen mainly in indicators that showed a higher score rather than the indicators that showed a lower score. This means that HMP in those indicators with lower score were basically identical among all study hospitals. These indicators include target balance, target setting and target sharing; continuous quality improvement; penalties on staff with dissatisfied performance; and rewarding the talents. Although these indicators are under the four different dimensions, the commonality is that they are all pertaining to the quality of care. This might explain why we did not observe any significant association of HMP overall score with quality of care. Clearly, these indicators are neglected in the current hospital management practices but are problems that all hospitals need to tackle with and should be addressed in the current health care reform in China. To be specific, we would suggest the central health authority to issue policies that require public hospitals to establish a quality of care continuous improvement system in each hospital. The system should have a clear governance structure that reaches from the top to the bottom managing roles, with clearly defined responsibilities of each role. The system should take regular actions to guide, inspect and evaluate the medical services for quality of care at both hospital and department levels, feed back the results, and follow up the rectification plan. The central government may also consider policies that give the director of public hospitals more power to better reward the talents and punish the staff with dissatisfied performance. Finally, the health authorities could provide the hospital directors training on hospital management, with target management emphasized in addition to general hospital management knowledge and skills.

In this study, target management performed the worst among the four dimensions of hospital management that we measured. As a modern management concept, target management refers to a management method which involves the active participation of all the hospital staff, in order to determine the work objectives from the top down and guarantee the goal realization from the bottom up. However, we found that participants were not familiar with the contents of their hospitals’ target. The importance of quality of care for hospitals seemed more like a well-known concept, rather than a guide of action. Considering some uncontrollable external factors such as dynamically changing policies and mass of imperative government orders, public hospitals might not be able to set up targets suitable for their own [20]. Few hospitals had actual action plan and measures to improve the quality of care.

We found that high management performance practices were significantly related to increased hospital total income, but when we adjusted for the number of beds and other variables, the relationship no longer existed. The results indicate that higher total income of hospitals with higher management score are actually due to more quantities of service that is reflected by hospital size rather than efficiency. This argument is also supported by our finding that the HMP score was not associated with cost per hospitalization.

There are some limitations in this study. First, although our study sample was a nation-wide county hospital sample, non-random sampling was determined by the design of the main study. Moreover, participating hospitals are all public county hospitals, of which characteristics may be different from those in cities. Thus, the study results should be interpreted with caution when generalized to all hospitals in China. Second, the sample size is small and may not be warrant for the insignificant correlations. Future studies are required to repeat our results. Third, our investigation at the department and staff level was focused on the cardiology department, the situation in other departments may not be well reflected. At last, to keep the HMP evaluation simple and feasible, our survey on HMP included only three different managing roles and one physician. It did not include other types of staff, such as nurses. Future studies may consider involving more roles, but the trade-off with the increase in work load should also be considered.

In conclusion, our study finds that the hospital management practice scored low in general and varied widely among the Chinese county hospitals. The hospitals with high and low HMP scores shared the common strengths and weakness in management practice. The commonality of weakness was the lacking indicators towards better quality of care and the commonality of strengths was the indicators towards more annual hospital total income. To be successful, the current healthcare reform in China should address the hospital management practices.

## Supplementary Information

**Additional file 1: Table S1.** The HMP rating framework and definitions for indicators and sub-indicators.

## Data Availability

The datasets analyzed are available from the corresponding author upon reasonable request. Contact wuyf@bjmu.edu.cn.

## Abbreviations

HMP: Hospital management practice
CPACS-3: The Third Phase of the Clinical Pathways for Acute Coronary Syndromes in China Study.
