# Supplementary material for: The hospital management practices in Chinese county hospitals and its association with quality of care, efficiency and finance
Source: BMC Health Serv Res. 2021 May 11;21:449. doi: 10.1186/s12913-021-06472-7 (PMC8111980; doi:10.1186/s12913-021-06472-7)
Supplement: Supplementary file 1 — Additional file 1: Table S1. The HMP rating framework and definitions for indicators and sub-indicators. [file 12913_2021_6472_MOESM1_ESM.docx]

**Table S1.** **The HMP rating framework and definitions for indicators and sub-indicators**

| **Dimensions** | **Indicators (definitions)** | **Sub-indicators** | **Definition of sub-indicators** |
| --- | --- | --- | --- |
| Target management | Target balance (The degree of hospital targets in their comprehensiveness and consistencies between long and short term, and between hospital and departments) | Comprehensiveness of targets | The comprehensiveness of specified targets in 4 aspects: finance, efficiency, quality of care and patient-centered services in hospital’s long-term and annual work plan |
|  |  | Consistency between short- and long-term targets | Consistency between long-term and annual targets in the above 4 aspects |
|  |  | Consistency between department plan and hospital plan | Consistency of between department and hospital plans in the description of targets in above 4 aspects |
|  | Target setting (The degree of appropriateness in setting hospital targets) | The bases for target setting | Defined by 1) the degree in accordance with the government requirements, hospital’s historical performance, performance of competitors, and consultation with senior staff; and 2) the degree of recognition of challenges by the hospital director in the 4 targets (Consistency between hospital challenges and annual plan) |
|  |  | Participation of hospital directors in department annual plan setting | Whether department and hospital jointly develop the department annual plan |
|  | Targets sharing (The degree of awareness and consensus on the hospital targets among all internal stakeholders) | Awareness of hospital long-term targets | Whether department directors and doctors are aware of hospital long-term targets |
|  |  | Awareness of hospital annual plan | Whether department director and doctor are aware of hospital annual targets |
|  |  | Identity of hospital long-term targets | The recognition degree of hospital long-term targets by department directors and doctors |
|  |  | Identity of department annual plan | The recognition degree of hospital annual targets by department directors and doctors |
|  | Target stretch (The degree in difficulty to achieve hospital’s, department’s and staff’s targets) | Target stretch | Degree in difficulty to achieve hospital’s, department’s and staff’s targets scored by department director and doctors |
| Operations management | Medical environment (The degree in space sufficiency and layout rationality) | Space sufficiency of outpatient area and inpatient ward area | Space sufficiency of outpatient waiting area and clinics, and inpatient ward space |
|  |  | The rationality of layout | The layout rationality of outpatient area and inpatient ward area |
|  | Layout of services (The degree of layout of services) | Distance from the gate to emergency room | Whether the distance is too far from the gate to emergency room |
|  |  | Distance between surgery ward and operating room | Whether the distance from surgery ward to operating room is too far |
|  |  | Placement of fall prevention signs | Whether the ward has placed fall prevention signs |
|  | Patient-centered services (The degree of convenience of services for patients) | Establishment of appointment register | Whether the hospital has established appointment registration for outpatient services |
|  |  | Establishment of "green channel" | Whether the hospital has established “green channel” for heart attack and other emergencies |
|  | Clinical pathway (The degree of standardization of services) | Availability | Whether the hospital and department has introduced clinical pathways |
|  |  | Facilitating measures | Whether the hospital has measures, e.g., staff training and electronic clinical pathway, to facilitate the implementation of clinical pathways |
|  |  | Perceived effects by responders | Evaluation of the effects of clinical pathway by staff at different administrative levels |
|  | Continuous quality improvement (The degree of continuous improving quality of care) | Quality management policy | Whether hospital has quality of care assurance policy documented |
|  |  | Governance | Whether hospital has governance system on quality of care, e.g., quality control committee/office/group |
|  |  | Specific measures of quality management | Specific actions taken as hospital’s quality management |
| Performance management | Hospital Performance appraisal (The degree of performance management at hospital level) | Hospital performance appraisal system | Whether hospital has a performance appraisal system for its departments and staff |
|  | Department performance appraisal (The degree of performance management at department level) | Criteria and publicity of performance appraisal | Whether hospital has assessment criteria for middle-level cadres and staff and publicize these criteria. |
|  |  | Contents of appraisal | The completeness of department performance appraisal system involving targets and work plan, department revenue, working disciplines, research projects and publications, quality of care, medical safety, talents program and patient satisfaction. |
|  |  | Appraisal of department directors | The completeness of the department director appraisal system involving public hearing, appraisal by committee, and appraisal by staff representatives |
|  |  | Discussion and follow-up of appraisal results | Whether appraisal results are regularly discussed on director’s meetings and followed-up by hospital director in time |
|  | Staff performance appraisal (The degree of performance management at individual staff level) | Contents of appraisal | The completeness of the staff performance appraisal system involving quantity indicators (workload such as number of patients in charge), quality indicators (patient satisfaction, medical errors, accidents and complaints), and others (research projects and publications, continuing education, working disciplines etc.) |
|  |  | Methods of appraisal | The completeness of the staff performance appraisal system involving establishment of department assessment group, assessment by department and assessment by peer staff |
|  |  | Discussion of appraisal results | Whether department regularly conduct staff assessment and discusses about the results on department meetings |
|  | Penalties on staff with dissatisfied performance (The degree of penalties) | Verbal criticism | Verbal criticism to dissatisfied performance, recognized by department director and doctors |
|  |  | Discussion of problems with staff | Department director’s discussion with the staff with dissatisfied performance, recognized by department director and doctors |
|  |  | Deduction of bonus | Deduction of bonus of the staff with dissatisfied performance, recognized by department director and doctors |
|  |  | Re-training | Re-training the staff with dissatisfied performance, recognized by department director and doctors |
|  |  | Remove from original post | Remove the staff with dissatisfied performance from original post, recognized by department director and doctors |
|  | Uneven workload (The degree of uneven workload) | Uneven workload between departments | Degree of uneven workload among departments, evaluated by department directors |
|  |  | Uneven workload within department | Degree of uneven workload within department, evaluated by doctors in the department |
| Talent management | Rewarding (The degree of rewarding high performance staff) | Bonus | Rewarding high-performance staff with bonus, recognized by department directors and doctors |
|  |  | Training opportunities | Rewarding high-performance staff with training opportunities, recognized by department directors and doctors |
|  |  | Promotion | Promoting high-performance staff, recognized by department directors and doctors |
|  |  | Spiritual incentives | Rewarding high-performance staff with spiritual incentives, e.g. verbal praise, recognized by department directors and doctors |
|  | Staff satisfaction (The degree of staff’s satisfaction with their salary, working environment and career development) | Salary | The degree of satisfaction of salary, evaluated by department directors and doctors |
|  |  | Working environment | The degree of satisfaction of working environment, evaluated by doctors |
|  |  | Career development | The degree of satisfaction on career development, evaluated by department directors and doctors |
|  | Attracting talent (The degree of the attractiveness of hospital talent program) | Talent recruitment | Existence of talent introduction system, recognized by hospital director and department directors |
|  |  | Attracting talents | The degree of hospital talent program attractiveness, evaluated by hospital director, department directors and doctors |
